# Enacting Culturally Relevant Pedagogy when “Mathematics Has No Color”: Epistemological Contradictions

**DOI:** 10.1007/s40753-023-00219-x

**Published:** 2023-05-30

**Authors:** Mollee Shultz, Eleanor Close, Jayson Nissen, Ben Van Dusen

**Affiliations:** 1 Physics Department, Texas State University, 601 University Dr, San Marcos, TX 78666 USA; 2 Nissen Education Research and Design, 2565 NW Lincoln Ave, Corvallis, OR 97330 USA; 3 School of Education, Iowa State University, Lagomarcino Hall, 901 Stange Road, Ames, IA 50011 USA

**Keywords:** Culturally relevant pedagogy, Identity, Epistemology, Hispanic-serving institutions

## Abstract

Culturally relevant pedagogy (CRP) seeks to improve equity in instruction and leverage students’ experiences by promoting academic success, cultural competence, and sociopolitical consciousness. We examine instructors’ perceptions of student identity to understand the ways undergraduate mathematics instructors are enacting or experiencing barriers to enacting CRP. Interviews with ten mathematics faculty at Hispanic-serving institutions identified two potential barriers to enacting CRP: first, instructors’ hesitance to communicate about student identity, especially with respect to race and gender; and second, instructors holding epistemologies that mathematics is culture-free. Despite these barriers, almost all interviewees implemented the academic success tenet of CRP. These barriers may prevent instruction around cultural competence and sociopolitical consciousness, which are the two tenets that most capitalize on students’ informal knowledge, identities, and cultural experiences. Changing discourse by taking more risks in conversation and inviting a more diverse range of people to the undergraduate mathematics community are potential ways to address these barriers.

## Introduction

Mathematics is a high-stakes academic subject in the United States school system due to its influence on social access (Schoenfeld, 2004), its impact on academic and economic opportunities (U.S. Department of Education, 2008), and its common misattribution of intelligence (Shah, 2013). At the same time, mathematics has a long history of systemically marginalizing groups of students and reinforcing racial hierarchies within and beyond the mathematics community (Larnell, 2016; Martin, 2019). For example, practices around who can and cannot enroll in mathematics courses based on placement exams and prerequisites create racialized barriers for academic achievement (Larnell, 2016). While trying to learn the content, Latinx and Black students have the additional burden of navigating hostile learning environments, such as coping with racial microaggressions (Hotchkins, 2016; McGee, 2016), stereotype threat (Good et al., 2008), and pressures to compromise their identity (Larnell, 2016). At times, they are characterized not only as uneducable, but also as troublemakers, criminals, and terrorists (Gholson & Martin, 2017).

Addressing these issues requires not only giving students equitable opportunities, but also shifting instruction to support the groups of students who have been harmed (Gholson & Robinson, 2019). Culturally relevant pedagogy (CRP; Ladson-Billings, 1995; 2006) provides a way to shift mathematics instruction to become more equitable. Ladson-Billings (2014) described CRP as instruction aimed to help students gain (1) *academic success*, “the intellectual growth that students experience as a result of classroom instruction and learning experiences”; (2) *cultural competence*, “the ability to help students appreciate and celebrate their cultures of origin while gaining knowledge of and fluency in at least one other culture”; and (3) *sociopolitical consciousness,* “the ability to take learning beyond the confines of the classroom using school knowledge and skills to identify, analyze, and solve real-world problems” (p. 75). CRP can guide educators to reinvent the mathematics curriculum to empower and play to the strengths of previously marginalized students. However, little is known about either the extent to which undergraduate mathematics instructors engage in practices consistent with these three tenets of CRP or what barriers exist for them to learn to do so.

Ladson-Billings (2006) explained that a commitment to teaching with CRP is essential to creating a society worth living in and giving students the education they deserve. When asked how to teach with CRP, Ladson-Billings explained she does not prescribe how to do so because it takes getting to know students and the issues relevant to their communities. She concluded by asking, “The question is not how can we do it but, rather, how can we not do it?” (p. 41). We agree that it is important, and we also see practical barriers for faculty and instructors that make implementing CRP a sizable undertaking. For example, to provide mathematical content that helps students appreciate their own cultures, instructors need to have a grasp of their students’ cultural backgrounds; this becomes more difficult as class sizes and course loads increase. Many of the institutions with student populations that could most benefit from CRP are the same institutions that lack resources to support faculty in learning and implementing new pedagogical practices. Minority-serving institutions are disproportionately two-year colleges (CCIHE, 2018), where instructors have higher teaching loads and fewer resources (Yuen, 2020).

To investigate these issues, we analyzed data from interviews with faculty from Hispanic-serving institutions (HSIs). In the United States, institutions with 25% or more full-time, Hispanic students can apply for HSI status (U.S. Department of Education, 2021). Instructors at HSIs are in a position to support a critical mass of underserved students, yet not much is known about whether they have the knowledge or resources to do so. In the past ten years, the number of HSIs has increased from 293 to 569 in the United States, a 94% increase (Burke, 2021). Their growing existence as a type of minority-serving institution seems to be circumstantial rather than strategically planned (Contreras et al., 2008). While some minority-serving institutions like historically Black colleges and universities are created to serve their student populations, HSIs are preexisting institutions that apply for HSI status in part due to their minority population. Although we focus on institutions in the United States, questions related to enacting culturally relevant pedagogy are relevant in any setting where there are social issues to think critically about or the student population is not entirely homogeneous.

With the rapid growth of HSIs, not much is known about how mathematics instructors at HSIs can respond to student needs. Hubbard and Stage (2009) found few differences in attitudes towards teaching and students between faculty at HSIs and faculty at predominantly White^1^ institutions. And while HSIs enroll large numbers of Latinx and Hispanic students, their patterns of unequal outcomes remain similar to the patterns at predominantly White institutions (Contreras et al., 2008; Laird et al., 2007). Cawley (2018) found that Latinx students in developmental mathematics courses who tended to accept classroom norms in traditional lecture-based courses were less likely to pass the course than students who pressed for change. These findings indicate that if instructors are not doing anything proactively to support their Latinx students, those students are starting with a disadvantage. Thus, it is worth investigating what, if anything, instructors are doing specifically to serve their student populations at HSIs, especially in the high-stakes context of mathematics classes. This article reviews the contexts of doing mathematics, some ways student identity has been researched in mathematics education, and culturally relevant pedagogy to give background for the present study.

## Literature Review

### Epistemology and Mathematics Background

STEM fields have historically been praised for the objectivity of the knowledge they produce. We are calling the idea that the validity of the knowledge is established independent of culture a *culture-free epistemology*. STEM fields including mathematics have historically been portrayed as culturally neutral subjects. For example, Traweek (1988) described the culture around doing physics as “an extreme culture of objectivity: a culture of no culture, which longs passionately for a world without loose ends, without temperament, gender, nationalism” (p. 162). The epistemologies of people that do mathematics may be seen as even more strict because the knowledge is produced by logic rather than grounded in experimental data. For example, it is still up for debate in the mathematics community if proofs done by computer simulation are true proofs (e.g., the Four-Color Theorem, Appel & Haken, 1977; Sümmermann et al., 2021; Tymoczko, 1979). In a study that included a range of STEM instructors, scientists from several fields noted that “mathematicians’ thinking is too abstract, context-free, or ungrounded in reality” (Rabin et al., 2021, p. 8). Our perspective is that the interplay between cultural context and the development of abstract mathematics is complementary and not separable. We agree with Ladson-Billings that mathematics is situated in cultural contexts. As Greer and Mukhopadhyay (2015) stated, “Far from being culturally neutral, mathematics [...] only makes sense when considered as embedded in historical, cultural, social and political - in short, human - contexts” (p. 261). Yet, it is understandable how instructors who have been enculturated into mathematics as being free of context would resist the idea of mathematics as being not only dependent on the real world, but also culturally-dependent.

Many mathematics departments also include mathematics education researchers with training and research in both mathematics and education research. Mathematics instructors with a mathematics background but not education background may have different beliefs and implementations of CRP than those with backgrounds in education research, based on their training or their original interest in their fields. CRP implementation is dependent on how students' identities are perceived, thus we turn to consider the work on student identity in mathematics education.

### Identity in Mathematics Education

Identity is increasingly a productive focus of investigation among mathematics education researchers (Darraugh, 2016; Graven & Heyd-Metzuyanim, 2019). Identity can serve to study the connection between mathematics learning and cultural activity (Sfard & Prusak 2005), acting as “a pivot between the social and individual” (Wenger, 1998, p. 145) facets of learning. Studies on identity in mathematics education have usually centered on the identities of students or the identities of instructors (Darraugh, 2016; Graven & Heyd-Metzuyanim, 2019). Notably absent from these studies are examinations of how instructors perceive student identity or use their interpretations of student identity to influence their instructional decisions.

We will outline some of the ways researchers have conceptualized identity as a proxy for how instructors might be conceptualizing their students’ identities. Two common themes in mathematics education identity literature are race and gender. Researchers have shown that students experienced gendered (review by Leder, 2019; Leyva, 2017) and racialized identities in their mathematics learning (Gholson & Robinson, 2019; Gholson & Wilkes, 2017; Hotchkins, 2016; Larnell, 2016; Leyva et al., 2021; Martin, 2019; McGee, 2015; McGee et al., 2017). For example, Larnell (2016) illustrated the narratives of two African American learners placed in non-credit-bearing remedial mathematics courses. Despite being high-achieving in their high school math trajectories, they chose paths that were just good enough or “satisficing” in college, perhaps due to not understanding the stakes of enrolling in remedial courses. Part of why this context matters in the U.S. is because remedial mathematics courses still cost the students full tuition, while getting them no closer to earning the credits necessary to achieve a postsecondary credential. Outside this U.S. context, these findings apply to any context where race matters. the students also questioned their own identities as they noticed the racial identities of who was enrolled in which courses and what potential implications related to intelligence that might have.

Relevant to instructors at HSIs, Rincón and Rodriguez (2021) showed that Latinx students drew from six different categories of cultural wealth to facilitate their persistence in STEM. Meanwhile, researchers have found that Hispanic and Latinx students in STEM fields experience imposter syndrome and microaggressions related to their racial identities (Chakraverty, 2022). More generally, there is awareness that the field of mathematics has harmed Latinx students with inequitable learning experiences (Association of Mathematics Teacher Educators, 2017).

Instructors can think of these identities separately or as part of students’ intersectional identities. To think of the identities as intersectional means to think of two or more identities simultaneously and how that combination of identities plays out in various settings (Delgado & Stefancic, 2017; Collins, 2015; Crenshaw, 1990). Pugach et al. (2019) conducted a review of the teacher education literature on how researchers addressed students’ and teacher candidates’ markers of identity. They found that researchers rarely recognized students’ or teachers’ multiple, intersecting identities and usually addressed identities as unidimensional. Ong et al. (2011) showed how the intersectionality of race and gender created unique challenges for women of color as they faced racism and sexism in their postsecondary STEM experiences. Studies of women of color have also shown how they capitalize on their lived experiences in creative and productive ways to make sense of mathematics (e.g., Adiredja & Zandieh, 2020). Instructors need to communicate about race and gender to access and leverage that informal knowledge.

### Implementation of Culturally Relevant Pedagogy

Considering the complexity of student identity, the implementation of culturally relevant pedagogy as customized to individual students is not simple. Ladson-Billings (2006) outlined the three tenets of CRP -- *academic success, cultural competence,* and *sociopolitical consciousness* -- without practical instructions for how to enact them. The first tenant of *Academic Success* pertains to enabling student academic achievement regardless of social inequities and hostile classroom environments (Ladson-Billings, 2006). Ladson-Billings (2014) and others (e.g., Nolan & Keazer, 2021) stress the importance of *cultural competence* and *sociopolitical consciousness*, because the goal is not just making the dominant version of mathematics more accessible, but rather changing what mathematics is taught. Ladson-Billings (2006) explained that she does not want to say how to enact them, though, because the way they should be enacted depends on the individual students with their unique contexts and backgrounds. CRP does not describe a list of practices or tasks that can be added to current teaching, but rather an approach that guides all aspects of instruction (Nolan & Keazer, 2021). To complicate matters, most studies of CRP have been conducted with racially homogeneous classrooms, for example, “All African American classes, all Latino/a classes, and so forth” (Morrison et al., 2008, p. 443). Student populations at HSIs often have heterogeneous racial compositions, which is challenging because Ladson-Billings (2006) called for understanding the backgrounds and cultures of each student for authentic enactment of CRP.

The K-12 mathematics education literature has reflected a number of efforts to incorporate CRP. Some pieces that seem common to many of the empirical implementations of CRP are strengthening and holding high standards for critical mathematical thinking skills (Bonner, 2014; Gustein et al., 1997; Gustein, 2016; Matthews, 2003), building on students’ informal funds of knowledge or cultural capital (Gustein et al., 1997; Gustien, 2016; Matthews, 2003), and developing students’ critical consciousness (Gustein, 2016), analogous to the three tenets Ladson-Billings originally outlined. Another common theme was building relationships with students (Bonner & Adams, 2012; Matthews, 2003). Studies such as the Algebra Project have found that student performance and affect were greatly improved by multicultural curriculum (e.g., Moses and Cobb, 2002).

In contrast to undergraduate contexts, instructors of K-12 courses who implement CRP usually have some flexibility with the mathematical content they need to include in their course, and their students are from relatively homogenous backgrounds because they grow up neighboring the school. Still, undergraduate mathematics educators are finding ways to make their courses more culturally relevant. While some studies focus on the academic success tenet (e.g., Moore, 2005), others incorporate topics that use mathematics as a tool to examine problems with cultural and political relevance to students*.* For example, researchers have embraced the implementation of CRP through a Funds of Knowledge approach (Anahalt et al., 2018; Jett, 2013; Voigt et al., 2020), building on the cultural and lived experiences students bring to the classroom. In a flipped calculus course, Voigt et al. (2020) explained that contextualizing problems in relevant ways was a mental tool that enabled students to navigate the transition from the real world to the symbolic world. They used geographic tasks that relied on local landmarks and issues of climate change. They also contextualized inquiry problems like the Magic Carpet Ride that reinvent concepts of span, linear independence, and linear dependence using local geographic landmarks (Wawro et al., 2012).

Anahalt et al. (2018) explained that one way students can build *cultural competence* and *sociopolitical consciousness* is through the mathematical modeling of issues in their own communities. Jett (2013) reported doing this in his own undergraduate mathematics classes by contextualizing mathematical problems to the cultural needs of African American students. Mgonja (2021) reported on the impact of culturally relevant learning modules that included topics like modeling interracial marriages, neighborhood gentrification, prevalence of mental health issues, and the impact of legalization of marijuana on number of arrests over time with systems of linear equalities, inequalities, and absolute value equations. He found that the Students of Color in his study outperformed White participants in learning gains. Examples like these show that mathematics is useful as a tool to interrogate social issues relevant to students. Yet, these issues are not typically included in the undergraduate mathematics course of study.

## Theoretical Framework

To advocate that instructors ought to adopt more culturally relevant practices, we need to develop an understanding of what instructors think culturally relevant practices entail. We examine instructors’ perceptions of student identity to understand what students they would be trying to serve with such change and theorize about what tensions could arise.

### Perceived Student Identity as a Lens for CRP

**Fig. 1:**
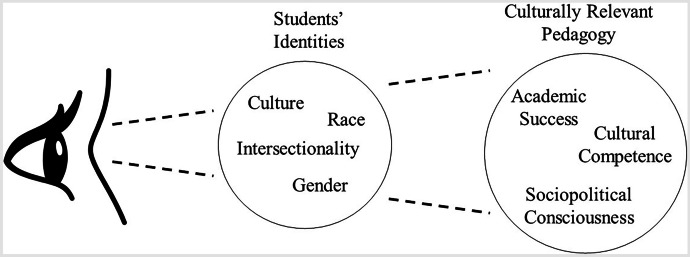
Instructors' perceptions of students’ identities acting as a lens through which they perceive culturally relevant pedagogy

Following Sfard and Prusak’s (2005) idea that identity can serve as a way to study the connection between mathematics learning and cultural activity, we conceptualize instructors’ perceptions of student identities as a lens through which they perceive instructional decisions (see Figure 1). The mathematics education literature, however, does not include a conceptualization for how instructors view student identity (Darragh, 2016; Graven & Heyd-Metzuyanim, 2019). Likewise, little has been published in undergraduate mathematics education on how instructors communicate about identity. Instructors’ hold beliefs internally that guide how they react to their perceptions of what exists externally. How instructors view their students’ race, gender, ethnicity, sexual orientation, and the intersection of these identities acts as a lens for what they see as being culturally relevant to those students. The instructors then decide how to package the mathematical content.

In a study where students chose their own culturally relevant projects, Enyedy et al. (2011) showed how students’ identities and informal knowledge was central to their choice. If instructors are doing the choosing, they are forced to make these inferences based on what they have learned about students’ identities. In studies of teachers noticing, teacher’s inferences can be deficit-based or their inferences can actively challenge deficit discourses (Louie et al., 2021). When enacting CRP teachers notice students’ assets and then leverage those assets to support student learning. For example, in Aguirre and Zavala’s (2013) tool for discussing the implementation of CRP in mathematical lessons, they include guiding questions such as, “How does my lesson help students connect mathematics with relevant/authentic situations in their lives?” and “How does my lesson support students’ use of mathematics to understand, critique, and change an important equity or social justice issue in their lives?” (p. 169). How instructors view students and the issues relevant to students’ lives shapes the way they can communicate and address these questions.

We suspect that epistemological beliefs surrounding mathematical content shape whether instructors even feel the need to notice students’ identities and be responsive to them. Given this framing, the research questions are as follows:

1. What barriers to enacting CRP arise for undergraduate mathematics instructors at HSIs?
2. Are undergraduate mathematics instructors at HSIs enacting tenets of CRP despite these barriers? If so, what does this look like?

The framing of student identity as a lens for CRP enaction gives a way of understanding how instructors’ beliefs, perceptions of student identity, and teaching decisions interact. The literature addresses potential tensions between beliefs and pedagogy, without the link of student identity.

### Tensions in CRP Implementation

Researchers have found a relationship between educators’ beliefs about the nature of disciplinary knowledge and their beliefs about teaching and learning that knowledge (Beswick, 2012; Cross, 2009; Ernest, 1989; Johnson, 2011; Martínez-Sierra et al., 2020). Love and Kruger (2005) found that instructors’ positive orientations around CRP correlated with higher student achievement, and Gay (2002) argued that the beliefs of teachers, such as their own critical consciousness, are important for providing learning opportunities for students who are not in the ethnic majority. The literature showed that some epistemological beliefs involved in doing “pure” mathematics set up instructors to perceive the mathematics as culture-free. However, it is possible for people to teach in ways that contradict their beliefs about a discipline (Schoenfeld, 1988; Shultz, 2022; Webel & Platt, 2015). So while holding a culture-free epistemology might contradict with the principles behind enacting CRP, instructors may still enact CRP. That is, it remains a question whether holding a culture-free epistemology about mathematics acts as a barrier to teaching in culturally relevant ways.

The literature has shown traces of this tension between the nature of mathematical reasoning and enacting the principles of CRP. Enyedy and Mukhopadhyay (2007) showed how facilitating productive mathematical discussions about a culturally relevant project was difficult because the discussions primarily highlighted the social and personal relevance of the projects rather than the mathematical content. In a project that involved the incorporation of culturally responsive active learning practices by a university’s mathematics department, Ellington et al. (2021) found that there was a tension between mathematics instructors being “right” and culturally responsive. Instructors' frequent perception of feedback on their pedagogy as a critique of their understanding of mathematical knowledge rather than of how they were teaching it required the researchers to adapt the feedback to minimize instructors' defensiveness. The mathematics instructors were concerned that students know how to arrive at correct solutions and have the essential skills needed to succeed; their focus on this concern often came at the cost of being responsive to students' reasoning that might lead the discussion “off track”. These are examples of how beliefs did not align with practice.

## Methods

### Sample

This investigation was part of a larger study that included 40 interviews with instructors from 27 HSIs, across a variety of disciplines -- 17 from biology, 7 from chemistry, 6 from physics, and 10 from mathematics. Participants were recruited using a stratified sampling technique to reach instructors from a wide variety of HSIs. Instructors were from a range of institutional categories (associate’s=11, bachelors'=5, master’s=8, doctoral=16) and locations (Southwest=20, West=10, Midwest=3, Southeast=2, East=2, Puerto Rico=3), and held a range of self-described identities of gender (women=15, men=25) and race (Asian=2, Caucasian=5, Hispanic=5, Indian=1, Latina=1, Latin White=1, Puerto Rican=1, South Korean=1, Turkish=1, White=22). We included participants from a wide range of academic positions. Eleven of the 40 participants were in non tenure track positions; 3 of 10 from mathematics. We interviewed both tenure-track and non-tenure-track faculty in our analysis in order to include a range of relevant experiences in our analysis. Non-tenure track faculty were under-represented in our study relative to their prevalence in higher education: 62% of all instructional positions at U.S. institutions are filled by part-time instructors or full-time non-tenure-track instructors (AAUP, 2018).

This paper focuses primarily on the 10 mathematics instructors, whose information is detailed in Table 1. Three of these participants did mathematics education research. The instructors represent a broad range of positions in varied types of institutions. Our investigation was qualitative, so our claims are not predictive; instead they illustrate possible implementations or barriers to implementation of CRP. We do not have more demographic details about instructors’ classroom makeup beyond the HSI status and regional location of their institutions. Our interviews did not prompt instructors to discuss specific demographic details of their courses, in order to minimize the extent to which the interview prompts and format shaped their discussion of student identity.

**Table 1:** Demographics, position, institution Carnegie classification, location, and field of each participant

Pseudonym	Self-identified demographics	Position	Carnegie classification	Region	Field
Denita	Latina woman	Full Professor	Doctoral University: High Research Activity	Southwest	Mathematics
Francesca	White woman	Adjunct Professor	Associate's Colleges: High Transfer-Mixed Traditional/Nontraditional	West	Mathematics
Kyung Joon	South Korean man	Assistant Professor	Special Focus Two-Year: Other Fields	Southwest	Mathematics
Matthew	White man	Associate Professor	Associate's Colleges: Mixed Transfer/Career & Technical-High Nontraditional	Southwest	Mathematics Education
Niya	Indian woman	Lecturer	Doctoral University: High Research Activity	Southwest	Mathematics
Samuel	White man	Assistant Professor	Doctoral University: High Research Activity	Southwest	Mathematics Education
Tiffany	Caucasian woman	Full Professor	Associate's Colleges: Mixed Transfer/Career & Technical-High Nontraditional	Midwest	Mathematics
Timur	Caucasian man	Adjunct Professor	Baccalaureate/Associate's Colleges: Associate's Dominant	Southeast	Mathematics
Theresa	White woman	Associate Professor	Doctoral University: High Research Activity	Southwest	Mathematics Education
Tomas	White, Latino man	Visiting Professor	Doctoral University: Highest Research Activity	Southeast	Mathematics

### Interview Protocol

The interview protocol was developed by the STEM Equity Project (www.stemequity.net). The protocol was piloted with eight STEM instructors by three of the authors and revised before it was used for the 40 participants. The first author conducted the one-hour, semi-structured interviews in Zoom and transcribed them using the automated service otter.ai. When the transcripts were unclear, often due to the automation not being familiar with bilingual participants’ pronunciations, interviews were transcribed using www.rev.com.

Participants were first asked to think of a course they teach most often or have taught for the longest amount of time. The data for this study arose primarily from responses to the two questions, “Do the identities of students who enroll in that course influence your approach or the way you teach it? If so, how?” and, “How would you describe the culture or climate for students in your department in terms of supporting their identities?” If participants asked for more clarification about what was meant by identity, the interviewer said, “For example, race, gender, being a parent, business owner, first generation, etc.” Some of the data also came from participants’ general descriptions of their own teaching practice and responses to a question that asked if “needing to address or accommodate the needs of students” or “concern for maintaining a good or inclusive classroom environment” resonates with them as a reason for making changes in their course. Instructors were never asked directly if they use culturally responsive pedagogy, nor were they asked directly what their beliefs are about the nature of mathematical knowledge. Yet, the questions about identity triggered responses related to these issues.

### Coding

All four authors collaborated to create and test the six codes listed in Table 2. Initial codes for culturally relevant pedagogy were taken from Ladson-Billings (2014) and were developed to answer the research questions. The remaining three codes emerged as we identified text that was especially explanatory (e.g., *Identity: YES* often explained why an instructor implemented one of the three tenants) or unexpected (e.g., when someone made comments about math being culture-free yet incorporated a culturally relevant practice). These last three codes covered the explanations and contradictions in the text that we had flagged. We did not strictly define what we counted as identity, but rather looked to instructors’ responses to understand what they were thinking about when they perceived students’ identities. As codes were applied, we further refined the descriptions to better account for the examples in the data. We coded examples of cultural competence and sociopolitical consciousness generously because they were uncommon, and we wanted examples of what instructors are doing.

**Table 2:** Code names, descriptions, and kappa scores (Cohen, 1960), n=40

Code	Description	% Agreement	*κ*
Academic Success	Expresses the intent or willingness to take into account students’ backgrounds, cultures or identity to set them up for academic achievement.	82.2%	0.63
Cultural Competence	Expresses the intent or willingness to set students up to understand things relevant to their own or other students’ cultures.	93.3%	0.37
Sociopolitical Consciousness	Expresses the intent or willingness to help students use the discipline to develop the skills to critically “identify, analyze, and solve real-world problems” that tie to sociopolitical issues that directly impact the students being taught. This can include bringing up issues that are controversial.	100%	undefined
Culture-free epistemology	Evidence that they believe that culture is somehow absent or neutral in mathematics.	93.5%	0.66
Identity: YES	There exists evidence that they intend or would like to consider student identity in their teaching practice.	50.2%	0.42
Identity: NO	Considers students' identity as not playing a role in their practice.	84.4%	0.61

To establish interrater reliability, the first author coded the full set of 40 interviews and the other three authors collectively acted as a second coder, each coding roughly one third of the interviews. Codes were compared on responses to the two questions stated above on identity for 50% of the full set of interviews. We report the percent agreement and kappa scores in Table 2. Kappa scores are useful because, unlike percent agreement, they account for agreement by chance (Viera & Garett, 2005). Agreement for Cohen’s kappa coefficient is considered fair for ranges .21-.40, moderate for .41-.60, substantial for .61-.80, and almost perfect for .81-1.00 (Landis & Koch, 1977), where 0 indicates agreement equivalent to chance and 1 indicates perfect agreement (Viera & Garett, 2005). Scores were at least fair for all codes except *sociopolitical consciousness*, which was undefined because there was only one instance.

After the data were coded, we used a spreadsheet to display each instructor, their demographics, and the number of instances of each code in their responses. We looked for patterns and relationships around who was implementing the three tenants, their personal backgrounds and contexts, and instances of the culture-free and identity codes. We noted that many instructors had contradictory codes. For example, we did not expect that instructors who alluded to holding a culture-free epistemology would report implementing culturally relevant practices. We also did not expect that instructors who explained they did not consider students’ identity as playing a role in their practice would also mention several aspects of student identity that affect their teaching. This caused us to summarize what culturally relevant practices instructors were naming, and then investigate how those were interacting with the codes for culture-free epistemology and identity yes or no.

For example, Tomas said he both did and did not attend to student identity, and incorporated one instance of CRP. He said that nationality did not matter (“It’s not important if the student is from Latin American, Australia, China, or Russia”), which was coded as *Identity: NO*. When asked if the identities of students who enroll in his math courses influence the way he teaches it, he said no, which was also coded as *Identity: NO.* However, he also said, “There are usually more men than womens in mathematics. …We need to find a way to make mathematics more active to womens.” He also talked about wanting to honor black women in the field by showing Hidden Figures in his class, which we coded as *Identity: YES*. While he said student identity did not influence his teaching, he also made an effort (however small) to encourage the women and students of color in his classes to see themselves and their challenges represented, which we counted as an example of the *Cultural Competence* tenet.

Kyung Joon is an example of an instructor who professed to not attend to student identity, thought math was independent of culture, and still implemented CRP. When asked if student identity played a role in his teaching, he said “I thought about that a lot. Because a lot of people actually asked me that question, too. Honestly, I don’t think I see that much difference in math. […] To me, it’s not really that relevant in math courses.” This was coded as *Identity: NO.* He went on to say, “Some professor’s trying just too hard using all different examples to cover a variety of cultures. To me, that’s actually more insulting, and sometimes, it’s kind of strange. I don’t know. I think science is more neutral in that sense,” which we coded as *culture-free epistemology.* At the same time, he adjusted his teaching for his students in a way that we coded as enacting *Academic success*. He said, “Here we have a lot of students that are really financially struggling, and they didn’t really go to the good high school.” He contrasted his current students with students at another university he taught at, where families were better off economically. For his current students, he said that he told them, “You don’t remember this kind of thing and that’s okay. We’ll review, but you have to catch up.” He continued, “I don’t blame them, but I review pretty much everything. Try to make them actually have those foundations as much as possible.” So his thoughts about mathematics being culturally neutral did not prevent Kyung Joon from reviewing the content in a way that made it accessible to students that did not have sufficient preparation.

What becomes notable in our results is how instructors’ ways of recognizing student identity are closely related to what tenets of CRP they enact We noticed that the contradictions we initially noticed were not necessarily contradictions, but rather they tended to correspond with the absence of the second and third CRP tenants (see section 6.3). We returned to the specific instances of the text to dig further into what aspects of identity were being named and which were not. With these analyses, we started to see a clearer picture around what issues were barriers to the rare implementations of the second and third tenants of CRP. Simultaneously, these codes revealed what was *not* a barrier to enacting the first tenant of CRP.

## Positionality Statements

The individual identities of the authors influenced our interpretations of the data and the theoretical framings we prioritized. Therefore, we include a positionality statement for each author.

Mollee Shultz: I identify as a Chinese, cisgendered, heterosexual woman raised with the privileges of being adopted into a White family. I have an M.S. in mathematics and a Ph.D. in mathematics education. I have taught as an adjunct instructor at three two-year HSIs. Remembering the limited time and access to expertise about things such as CRP as an instructor, I am motivated to understand how to use my current position to make it more accessible to others.

Eleanor Close: I identify as a White, cisgendered, heterosexual woman. I hold B.A. and M.S. degrees in physics and an Ed.D. in Curriculum and Instruction. I am currently an associate professor of physics at a regional HSI; previously I taught for 8 years at a small liberal arts university and for three years as a high school science teacher. These experiences, combined with current work supporting instructional change efforts across multiple STEM departments, have motivated me to understand the structures, beliefs, and relationships that shape STEM instruction and classroom interactions at HSIs.

Jayson Nissen: Identifying as a White, cisgendered, heterosexual man provides me with opportunities denied to others in American society. My experience growing up poor and serving in the all-male submarine service motivated me to reflect on and work to dismantle oppressive power structures in science. I brought perspective to this work on identity that was shaped by my having a PhD in physics, doing education research and being a White man.

Ben Van Dusen: I identify as a cis-gender, heterosexual White man. I have a B.A. in physics, M.Ed. in Educational Leadership, and a Ph.D. in Science Education. Prior to earning my Ph.D. I worked as a high school science and mathematics teacher. When this research project began, I was faculty in a science education department at a teaching-intensive HSI. I am now an education faculty member at a research-intensive institution. My motivation to engage in this project comes from my experiences teaching science courses at HSIs and my ongoing work preparing future K-12 science educators to create a more just society.

## Results

### Barrier 1: Communication about Racialized and Gendered Identities

Many mathematics instructors (7 of 10) indicated they took students’ identities into account in their teaching. Table 3 details how many instructors expressed identity playing a role in their teaching, not playing a role in their teaching, both playing and not playing a role in their teaching, or not expressing anything about student identity shaping their teaching. The kinds of identities they named as influencing their teaching included age, caregiver status, ethnicity, first-generation status, gender, international student status, mathematics experience, race, socioeconomic status, and neighborhood. These were all ways instructors positioned students as belonging or not belonging to communities that influenced the students’ experience in the mathematics classroom, and thus influenced how they taught. On the other hand, many instructors (6 of 10) said that students’ identities did not influence the way they taught. The kinds of identities we heard instructors name as *not* influencing their teaching was a much shorter list: race, gender, and nationality. Given that all instructors in the study taught at HSIs, the tendency to claim students’ race did not inform instructional decisions is especially notable.

**Table 3:** Percentages (and raw numbers) of instructors who indicated student identity did or did not play a role in their teaching among math instructors and among the full sample of science and math instructors.

Evidence or explicit statement that	Mathematics (*n*=10)	Total (*n*=40)
(1) Student identity does play a role in teaching	70% (7)	88% (35)
(2) Student identity does not play a role in teaching	60% (6)	48% (19)
Both (1) and (2)	30% (3)	38% (15)
Neither	0%	3% (1)

Generally, instructors more readily named how less politically-charged labels influenced their teaching. Instructors seemed wary of making any statement that would imply that their teaching somehow discriminates based on gender, race, and nationality. For example, Tiffany said, “I think of them as individuals, but not necessarily as identities, if that makes sense. So given the fact as far as you know, their color, their gender or anything else, you know, it’s all the same to me in a sense when I teach.” Niya said she did not differentiate offering support based on gender or race (she used some examples) but instead described giving extra support to a student based on their health issues. Some instructors expressed a desire to not come across as making assumptions about students.

Some instructors explained that student identity influenced their teaching, and that they considered student identity in a holistic way. For example, Samuel, a mathematics education researcher, explained that he took identity into account, but not in a straightforward way that assumed a certain type of teaching corresponded with a certain identity. He explained, “Things like race, gender identity, first-generation, they all sort of get boiled into building this relationship with the individual students. I don't want to treat all first-generation students as monolithic [...] assuming that because they're a first-generation student, they're gonna struggle with X,Y, Z.” He approached identity from the idea of caring for the whole student, and he put thought into how to learn about their lives outside the classroom. For instance, he had an assignment at the beginning of the course where students personified mathematics by creating a script, and he used those as entry points to getting to know his students.

At the same time, all three mathematics education researchers in the study (Theresa, Samuel, and Matthew) noted the importance of race, gender, or ethnicity. Only one non-education researcher (Denita) noted race. Theresa explained that race and gender were important issues less in terms of the mathematical content she covers, but more in terms of caring for them as underrepresented persons in mathematics. She said,

*My experience with the course has been that I tend to have very few, relatively speaking, students who have African American or Hispanic backgrounds. [...] And so I think quite a bit about whether they find themselves feeling alone in the class if the whole world of math, at a certain point, tends to look much more white and Asian. And so I have a consciousness about what I put them into groups, if their ideas are being heard, and if they’re feeling comfortable sharing their ideas.*

She was noticing something about students’ identity (namely race) and being thoughtful about how it impacted their participation. Samuel described noticing his students’ demographics when he began work at an HSI: “Then my first class was maybe like 50%, white and 50%, Hispanic. So in education, there are still very few African American teacher candidates.” He went on to describe how this impacted his teaching. Likewise, Matthew mentioned that, “I have noticed some of the examples [...] can be a little bit insensitive in terms of gender, race or other types of things. So I do change out some of the different elements.”

We note the pattern of mathematics education researchers’ communication about race, gender, and ethnicity because the epistemological assumptions behind doing mathematics education research and mathematics (non-education) research are distinct. While mathematicians without an education background argue inductively to defend the truth of a claim, mathematics education researchers depend on a diverse variety of more empirically based methods. When Kjung Joon said that he did not consider student identity as influencing his teaching, he said, “especially some people from education backgrounds, they question that a lot,” indicating that he thought people with education backgrounds would think that student identity was important in a math classroom. Two of three mathematics education researchers in this study’s sample explained that they wanted their students to understand more than mathematical knowledge. Samuel explained why he had his future teachers work with manipulatives. He said,

*I don't tell the department people I'm doing this because they view it as a purely math course. [...] If you frame it like, this is what you went through as a kid. This is what you want your students to do, so I think it's valuable for you to go through the experience yourself.*

He is explaining a human experience he wants students to have and learn about. And further, he did not think the mathematics department aligned with his opinion. He continued, “And the math department didn’t do any of this. They just talked pure mathematics.” Similarly, Matthew related that the end of his statistics course is a project where students design a study. He said that, “my core assessment is to be able to see how they are able to employ techniques to the study.” Thus the knowledge education researchers see themselves teaching has a different scope than that taught by mathematicians without the education research background. In turn, that perceived scope is related to the perceived importance of things like race, gender, and ethnicity.

Some of the same instructors who explicitly stated that students’ identities did not play a role in their teaching also separately gave contradictory examples of doing so in the same interview (3 of 6), as enumerated in Table 3. While their initial responses reflected hesitance to come across as making assumptions, discriminating, or stereotyping, they showed that they put thought into responding to students’ identities in their teaching. For example, although Tiffany said she did not get to know students’ identities, she went on to explain how she pointed out to students who are not English-dominant speakers that they may have to do more work to decipher the contexts in statistics problems, and acknowledged that that is a disadvantage they had to handle. She may have held a different conception of identity which did not make this contradictory in her perspective. Tomas said he thought student identity did not play a role in how he taught because the problem was not that mathematics did not serve one identity type, but rather that it has “very bad public relations'' with everybody. He said that though he had heard things like needing to make mathematics more attractive to women, he felt he needed to make mathematics more attractive to everybody, regardless of gender. At the same time, he encouraged students in his class to watch Hidden Figures to be exposed to women doing mathematics.

As another example, Francesca initially stated “We don't see a color or sex or anything,” speaking with praise about the climate of her mathematics department towards supporting students’ identities. Shortly afterward, she stated:

*Different ethnicities have different minds. They're all very good at mathematics, but it's like they have flavors to them. And this is what I find so exciting about mathematics. Mathematics has an infinite number of sides to it, more than a diamond. And the way people understand is different. And when a student doesn't understand what you've said, it's an opportunity to rejoice with that student and then try another way.*

While rejecting the influence of race or sex on the climate of her mathematics department, Francesca prided herself in being able to teach in a way that responded to students’ ethnicities. Thus she and others who resisted communicating about student identities still taught in a way that was responsive to student identities. Identity still functioned as a lens for their instructional decisions.

### Barrier 2: Epistemologies that Mathematics is Culture-Free

Two mathematics instructors (of 10) indicated that mathematics was free of culture. We did not ask them directly about their epistemological beliefs and it is possible that more instructors held these beliefs without us knowing. Both of these instructors explained that there was some objective truth expressed by mathematics that was not culturally dependent. It seemed they were speaking more about reifications of mathematics than participation in doing mathematics, in that they talked more about mathematics as a thing rather than an activity that they did. Francesca said, “Mathematics has no color. Maybe that’s debated, but you look at something like the ocean and the color currents in it. And you know that those are all governed by mathematics. And many, many forces. And there’s just nothing about ethnicity in it.” She viewed mathematics as free of culture and that the knowledge generated through doing mathematics reflects objective rules in the world.

Kyung Joon similarly expressed that mathematics was free of cultural context. He stated, “Especially math, it's not really about your opinion, you get the wrong methods, then you get the wrong answer. It's not a bad thing. There's a kind of a right answer.” In addition to believing the knowledge produced by doing mathematics was culture-free, he also did not think the teaching and learning of mathematics was culture-dependent. He said that he did not think students’ identities or cultural backgrounds made a difference in teaching mathematics. He furthered that including some cultural customization might be harmful. He said, “If the math professor used K-pop as some kind of example, or some Korean barbecue, they wouldn't make the math problem easier or more understandable. To me it's not really that relevant in math courses.” He said that, “to me, that’s actually more insulting” than having no cultural references.

At the same time, he did not seem close-minded to learning how to teach in culturally relevant ways. He said he had attended conferences about teaching where they talked about this, but their teaching recommendations had always been for general courses. He said, “A lot of time when those people were talking about the different pedagogies, it wasn't about the science. It was about more general courses.” And even though those educators said it could be done in mathematics, Kyung Joon said, "No, you just cannot do that.” He expressed interest in attending similar training that specialized in mathematics teaching.

A third instructor, Theresa, explained that others in her department held this belief. She said, “Overwhelmingly, the department probably leans towards, ‘Math is math. Math is neutral. Math doesn't discriminate.’” She herself did not agree and she said there are others in her department who are very aware of issues of identity and its relation to the discipline, but she said, “by no means is it the majority”. She herself was a mathematics education researcher.

### So Did They Use Culturally Relevant Pedagogy Anyway?

Almost all mathematics instructors (9 of 10) reported using at least one of the CRP tenets, as shown in Table 4.^2^ Many instructors reported enacting at least one tenet of CRP even though they expressed one of the two barriers (resistance to communicating about student identity or culture-free beliefs), as shown in Table 5. While these instances initially seemed like contradictions, Table 5 demonstrates how the barriers correspond with the Cultural Competence and Sociopolitical Consciousness tenets. We outline themes and examples of how instructors enacted each tenet, and explore the seeming contradictions.

**Table 4:** The percentage (and number) of instructors in mathematics (n=10) and STEM (n=40) that enacted tenets of CRP

Evidence of the CRP tenet	Mathematics (*n*=10)	Total (*n*=40)
Academic Success	80% (8)	75% (30)
Cultural Competence	20% (2)	23% (9)
Sociopolitical Consciousness	10% (1)	3% (1)
None	10% (1)	10% (4)

**Table 5:** The percentage (and number) of mathematics instructors (n=10) that expressed enacting CRP, even after expressing one of the given barriers

	Barriers to enactment	
Evidence of CRP	Identity: NO (n=6)	Culture-free beliefs (n=2)
Academic Success (n=9)	83% (5)	100% (2)
Cultural Competence (n=2)	17% (1)	0% (0)
Sociopolitical Consciousness (n=1)	0% (0)	0% (0)

Most instructors (8 of 10) reported enacting *academic success*. Even instructors that expressed the identity (5 of 6) or culture-free (2 of 2) barriers gave instances of enacting this tenet. We thought of this tenet, see Table 2, as capturing practices where instructors clearly went out of their way to change their teaching to ensure the academic achievement of students based on their individual background, culture, or identities, often related to systemic inequities that students faced. Theresa explained that for African American or Hispanic students, she wanted them to not feel alone despite the reality that there are often relatively few of them in her classes. She tried to be attentive to how they were participating and making sure their voices were heard. To give a sense of what was coded as *academic success* and what was not, if Theresa had talked about wanting students to not feel alone but had not mentioned doing so on account of specific groups facing systemic inequities, we would not have coded it as *academic success.*

Some of the general examples of this in our sample, which we detail below, were speaking to students in Spanish (Matthew and Denita), altering the examples that appeared in textbooks to be more inclusive (Matthew), upholding high academic standards (Francesca), taking personal responsibility for students’ success (Francesca), and directing students to extra tutoring (Niya). Three instructors, Tiffany, Denita, and Samuel, mentioned trying to help students have access to the resources they need such as access to online materials and textbooks. During class, Kung Joon said he reviewed more material for students financially struggling or who went to a “bad high school”.

Instructors remarked that trouble accessing necessary resources like the internet was especially pronounced during the COVID-19 pandemic. Denita said,

*“I hope during the pandemic the faculty were more supportive. As a university, we talk more about being cognizant that not every student's internet might work. And so they might need extension on homework, because they had to work and some of the family members that may have lost jobs.”*

Similarly, Tiffany said, “Especially with COVID, and everything, I started recording my lectures during class, so then they can go back.” So a way instructors professed enacting *academic success* was through responding to challenges that became especially noticeable during the pandemic when full-time virtual learning was abruptly implemented.

These ways of supporting students’ academic success were named in connection to ways students were identified. For example, Denita said students were roughly 85% Latino, so responding in Spanish was important at times. She said many of her students were low income or dealing with lost jobs during the pandemic, and as a result needed extensions or had trouble with internet access. She also cited the low income of her students as being a reason why the entire community needed more open access textbooks.

Finally, Samuel mentioned a change in his perspective on assessment since beginning to teach at an HSI. He said that while teaching at a non-HSI in the past, students were similar. He said, “Every class I taught was always like, 21-year- old female, White, female, blonde hair, and had very similar backgrounds.”

In contrast, at his current institution, he said students in his class tended to be 50% Hispanic and many of them were parents. He changed his view of assessment as he tried to understand what his students might be dealing with outside the classroom. For example, instead of giving a low participation grade for a student that missed class, he would check in with them to see why they could not make it.

There were not many instances of *cultural competence* (2 of 10), which we defined as instructors’ intent to set students up to understand things relevant to their own or other students’ cultures. Instructors that expressed the identity (1 of 6) or culture-free (0 of 2) barriers mostly did not mention enacting this tenet. This pattern was also true in the larger sample that also included physics, chemistry and biology instructors.

An example of cultural competence we saw was instructors trying to give students a better sense of representation in the discipline, which was also a theme in the larger sample of instructors. This happened even when instructors said student identity did not influence their teaching. For example, Tomas, stated that student identity did not play a role in his teaching. Meanwhile, to better represent who did mathematics, Tomas tried to encourage students to watch Hidden Figures, a film that features four Black women mathematicians. The depth of this assignment seemed limited. He said he had one project where they were using something from the movie, but it did not seem like he showed the movie during class, gave students any form of credit for watching the movie, or discussed the racism and sexism portrayed in the movie. In fact, he said, “Students didn’t care at all about the movie. They only care about the final exam and how to get points.” When thinking about his effort to help women see themselves in math he remarked, “I tried to do something about it, but I believe that was not so great.” This example illustrates how instructors can hold two contradictory beliefs as Tomas also stated that student identity did not play a role in mathematics learning.

The other instance of *cultural competence* and the only instance of *sociopolitical consciousness* was from Samuel. He talked about students in his mathematics for future teachers class being interested in special education, and having students read an article about it. This was an example of trying to instill *cultural competence* because it was an attempt to help students understand how the content tied to understanding some other students’ (often inequitable) cultural experiences. We coded this as an instance of *sociopolitical consciousness* because it showed that Samuel was trying to help students use the discipline to develop the skills to critically “identify, analyze, and solve real-world problems” about mathematics learning and students with other learning needs. He remarked how this also included addressing misconceptions, such as students making comments about autism and mathematical ability due to drawing from pop culture examples like the movie Rain Man. More generally, he noted that he has students read articles in a course on equity that challenge students’ conceptions. He said, “We have one student who … ties it back to something that’s personal to him. [...] Trying to get him to realize that these things aren’t invalidating your thoughts, [but] are just taking a different point of view.” Again, he was attempting to help a future teacher understand and address different marginalized students’ experiences in their future mathematics teaching.

As shown in Table 5, holding culture-free beliefs or relating a hesitance to incorporate student identity into their teaching do not preclude instructors from incorporating practices associated with the academic success tenant. However, valuing student identity and culture appear to lead to more robust implementations of CRP. In the case of Samuel who explicitly stated, “I kind of come from the lens of building relationships with students as a way to tap into their cultural capital,” he was more inclined to incorporate practices associated with all three tenets of CRP. He explained that in his first year at an HSI, “I think I did a lot of work in my first year in terms of having to get to talk to students more. At [previous institution] It was kind of easy, because I had the same conversation starters. And I could just reuse, reuse, reuse. [...] Also getting to know more just about the culture of [state] in general, and weaving that into the classroom.” When asked what the impact of getting to know his students more deeply had on his instruction, he responded by explaining the practices that fell under the academic success tenet. That is, Samuel is an example of an instructor whose disposition around actively incorporating student identity in an asset-based way into his teaching led to authentic appearances of CRP.

## Discussion

As undergraduate mathematics instructors teach increasingly diverse populations at HSIs, they ideally adjust their instruction to serve those students. As reflected by the literature and shown by this study’s data, some faculty are grappling with whether or how CRP has a role in mathematics instruction. Due to some of the resistance professors have shown in the past to CRP (Ellington et al., 2021; Enyedy & Mukhopadhyay, 2007) this study investigated how this could be rooted in beliefs and perspectives perpetuated by the community. Investigating our first research question: *What barriers arise to enacting CRP for undergraduate mathematics instructors at HSIs?,* we found two potential barriers to the enactment of CRP. We discuss the connection between these barriers and CRP enaction and then the implications these results have for future work.

### Communicating About Race is a Critical Missing Link for CRP Enaction at HSIs

The first barrier was that, despite teaching at HSIs with diverse student populations, participants tended to not talk about student identity in the context of their instructional decision-making, especially with respect to race. This was especially true of instructors not involved in education research. That instructors were often incorporating considerations of student identity, but resisted communicating about it, has implications for how undergraduate mathematics instructors can grow responsive to student identity. Communication about identity matters because capitalizing on students’ identities and subcultures requires learning about them (Hill, 2006; Moschkovich, 2002). Not communicating about race contributes to the problem that racial differences in mathematics performance have a tendency to be depicted as effects, rather than the product of the racialized nature of students’ mathematical experiences (Martin, 2009). Part of the challenge in implementing CRP is negotiating what counts as relevant (Enyedy et al., 2011). In most empirical studies of CRP, the instructor decided what counts as culturally relevant. Implementing each tenet of CRP requires some recognition and communication about how to address student identity. When instructors discussed transforming their instructional practices in ways we interpreted as CRP, they did so based on their view of students’ identities and backgrounds (as illustrated in Fig. 2). For example, viewing students as diverse with nonbinary genders and racial identities influenced Matthew to alter examples in his course’s statistics textbook that seemed insensitive through that lens. As another example, recognizing that his teacher-students will have their own students with exceptional learning abilities caused Samuel to address issues of special education with his course for future teachers. Thus, instructors being hesitant to communicate about racial student identity makes it difficult for change to occur at a community level to address the needs of a growing Hispanic and Latinx population.

Our findings align with literature showing that communication around racial and ethnic identities are often purposefully avoided.In the era of social media, the wrong statement can lead to public shaming that can ruin careers in an instant (Ronson, 2016). Historically, lawmakers have avoided talking about race in legal documents in the U.S. under the guise of “color-blindness” (Annamma et al., 2017). Originally this was a term invoked to express that laws were to be implemented *regardless* of race, but instead it became a way to *evade* issues of race (Annamma et al., 2017). Annamma et al. (2017) contended that “color-evasiveness” is a preferable term to “color-blindness,” as the latter term equates the decision to ignore race to an unavoidable disability. Gutiérrez (2013) explained that using static cultural markers to infer students’ identities can lead to viewing students in a deficit manner. While it might not be good to use labels to define students in ways that trigger deficit thinking, it can be harmful to not mention racial identities at all (Larnell et al., 2016).

Our findings were also aligned with claims that instructors did not want to make assumptions about their students based on their appearance or labels. Some instructors saw student identity as complex. Kjung Joon, for example, did not want to essentialize students by tossing in shallow cultural references. Adding to the challenge, HSIs tend to be composed of many different historically marginalized groups (e.g., American Indian, Alaska Native, or Pacific Islander), so learning about students in authentic ways is not a simple ask for these instructors. The concern about making superficial cultural references is reflected in the literature. Celebrating holidays or speaking a few words in students’ native language can be well-intentioned but can heighten the “sense of otherness” experienced by minority students (Young, 2010, p. 252). However, the fear of making assumptions seemed to make it difficult to talk about race altogether for some instructors. Given the amount of research that shows learning mathematics is a racialized experience, and that this study was conducted with instructors teaching at HSIs, we saw this as a barrier for enacting *cultural competence* and *sociopolitical consciousness*.

Two instructors in our sample expressed the epistemological belief that mathematics is culture-free. It seemed like Francesca was mostly viewing the reifications of mathematics as culture-free. In her case, the duality of participation and reification for making meaning helped explain what initially seemed to contradict. Francesca noted that the process of understanding mathematics was culture dependent (“different ethnicities have different minds”) and yet the resulting mathematics was not culture dependent (“mathematics has no color”). Kyung Joon, on the other hand, saw both the process and the result as creating objective truth (“You get the wrong methods, then you get the wrong answer”). Generally, we think that in the framework of CRP, *academic success* has more to do with the process of learning mathematics, while *cultural competence* and *sociopolitical consciousness* require rethinking what is taught in standard mathematics courses. This interpretation aligns with the result that instructors provided examples of incorporating *academic success* but not of *cultural competence* and *sociopolitical consciousness.* The latter two tenets require more explicit grappling with student identity, which most instructors seemed hesitant to do.

The second research question was: *Are mathematics instructors at HSIs enacting tenets of CRP anyway? If so, what does it look like?* The results revealed that many instructors were still able to enact *academic success,* despite being resistant to communicating about identity and holding culture-free beliefs about the nature of mathematical knowledge*.* However*,* this study empirically illustrates Ladson-Billing’s (2014) concern that the second and especially the third tenets of CRP are not implemented in practice. *Cultural competence* and *sociopolitical consciousness* are key aspects of CRP contributing to identity development and greater social change, so it is concerning that their enactment was so rare. So what can the mathematics community do to overcome these barriers? First, we do not want to downplay the importance of *academic success*. Enabling historically marginalized students to succeed in the current mathematics system by customizing instruction to their unique strengths and needs is valuable. We see it as encouraging that instructors implement *academic success* while these barriers exist. Still, showing how mathematics is a powerful tool with cultural relevance and that can be leveraged for the political issues relevant to students is an essential step for transforming mathematics to include these students. We offer some ideas for future research that address the second two tenets.

### Future Directions for Enacting CRP

The barriers identified in this paper can serve as a guide to where people and departments can push to gain traction around culturally relevant practices. Henderson et al. (2011) found that effective change strategies in undergraduate STEM departments align with or change faculty beliefs, include long-term interventions, and recognize the institution as a complex system. We outline three recommendations that consider Henderson’s findings as applied to increasing the enactment of CRP, two around increasing the communication around students’ racial identities, and the other around addressing the demands of faculty as they work in complex institutions. 

First, the discourse around racialized aspects of student identity can shift at the individual level. Dr. Loretta Ross advocates for calling people in instead of calling them out (Bennett, 2020). In the current call-out culture, when “people publicly shame each other online, at the office, in classrooms, or anywhere humans have beef with one another” (Ross, 2019, p. 11), people are shamed and alienated in a toxic way. The person doing the calling-out gets to show their superior understanding of racism instead of doing the more difficult work of changing the system. Ross says it would be more productive to use the conversation as a learning opportunity by reaching out privately and discussing the issue one-on-one. While the current combination of culture-free epistemological beliefs and fear of essentializing students might lead instructors to avoid discussions of race, individuals can counter this in the way they converse with colleagues. How instructors perceive the roles of students' multiple identities in their courses and how instructors decide to respond to their perceptions is a direction for future research.

Second, the literature suggests that one method for mathematics departments to transform their cultural practices and increase the enactment of CRP is through the diversity of people with contrasting experiences that they invite into their professional communities. Henderson et al. (2011) found that an ineffective approach to institutional change is a “top-down” process where a department tries to change instruction with policy-making. The flipside of this is changing the composition of the faculty so that differing beliefs will infiltrate the community.

The data in this study suggested that CRP enactment might happen by welcoming and interacting with mathematics education researchers or other education researchers who model taking risks around identity conversations and culturally relevant mathematics content. For example, a mathematics instructor with training from an education department, such as Samuel, can bring in ideas from the education community. Indeed, one of the instructors from the larger sample remarked, “We hired our first physics education faculty. And I was very worried at that time. I said, ‘Yeah, you hire somebody that’s so different from all the other faculty. He or she will have a difficult time.’ [...] [But then Educator 1 and Educator 2] started showing all those cool tools that you can use to teach and I’m enough of a teacher to appreciate that.” The education researchers in our sample talked about race, and they had a wider range of vocabulary in expressing student identity and how they saw it playing a role in their instruction. A mathematics department needs the language and skills to discuss race if it hopes to address racism. While this evidence comes from a small sample size, we pose that this study provides preliminary indications that the presence of education researchers in the department can change the trajectory of the department around beliefs that prevent action. 

Another way departments can think about increasing communication about race is by hiring racially diverse faculty. In our dataset, the only non-mathematics education researchers who talked about race as an aspect of student identity that played a role in their teaching were Tomas and Denita, who themselves were Latino and Latina. Though we do not claim that identifying as a person of color leads to the ability to communicate about race, a diversity of lived experiences within a multi-racial community of practice lends itself to conversations about race. Changing the composition of STEM departments could be a long-term intervention to change the epistemological beliefs identified by this study – that participation in learning and doing mathematics is culture-free.

Finally, considering Henderson et al.’s (2011) recommendation to consider faculty as working in complex institutional systems, many of the faculty or adjunct instructors at HSIs have many demands with their positions and not much time. To do the difficult work of learning to communicate about identity and exploring belief systems, instructors need time and incentives. As a community college chair, Tiffany was teaching seven sections during the semester she participated in this study. Timur, an adjunct faculty, usually teaches eight to ten courses per semester. Both Tiffany and Kyung Joon stressed that they had attended professional development about making instruction culturally relevant, but did not see the connection with the mathematics content. Danita explained that in her department, they are told teaching is important but then publishing is what is evaluated. HSIs enroll large numbers of students who would benefit from CRP. The faculty at many HSIs, however, have little additional time and energy to create and implement CRP. While Ladson-Billings (2014) argues that an exact prescription of how to implement CRP undermines the reflective practice integral to CRP, instructors do not have the resources or expertise to invent entire curricula from scratch on their own as they get to know each cohort of students. Development of practical applications of CRP to common undergraduate mathematics courses like the calculus series, and the dissemination of the materials that already exist (such as those being created as part of the SEMINAL project, e.g., Ellington et al., 2021) can support more instructors in using CRP and engaging in the reflective practice that CRP supports.

## Conclusion

This study offers theoretical framing for understanding how instructors make meaning about student identity, and how it can help or hinder CRP implementation. The use of perceived student identity as a theoretical lens can help researchers better understand how instructors implement CRP or other equity-focused instructional practices. This study gives preliminary evidence that instructors’ epistemological beliefs are related to their ideas about whether student identity and culture is relevant to doing and learning mathematics and to the content of mathematics itself. Most instructors in our study avoided discussing students’ racial and ethnic identities or described these identities as irrelevant to mathematical teaching and learning; both of these stances create barriers for enacting the *cultural competence* and *sociopolitical consciousness* tenets of CRP. Our findings suggest that the hiring of faculty with more experience communicating about race and the development of culturally relevant tasks for core undergraduate mathematics courses are two practical routes for instigating long-term change.

## Footnotes

1 In this publication, we capitalize all races, including White, emphasizing that there is no default race and that they are all social constructs with associated sets of cultural practices.

2 We consider implementing *academic success* alone as not sufficient to be implementing CRP in a complete sense because Ladson-Billings (2014) emphasizes the tenets of *cultural competence* and* critical consciousness.*
